# Ethical, legal, and sociocultural considerations in neural device explantation: a systematic review

**DOI:** 10.3389/fnins.2025.1568800

**Published:** 2025-11-03

**Authors:** Manuela Vooijs, Katherine Bassil, Anne van den Brink, Odile C. van Stuijvenberg, Nick F. Ramsey, Karin R. Jongsma

**Affiliations:** ^1^VU University Amsterdam, Amsterdam UMC, VU Medical Center, Amsterdam, Netherlands; ^2^Bioethics & Health Humanities, Julius Center, UMC Utrecht, Utrecht, Netherlands; ^3^Department of Neurology and Neurosurgery, Brain Center, University Medical Center Utrecht, Utrecht, Netherlands

**Keywords:** neurodevices, neural implants, brain-computer interfaces, explantation, ethics, research ethics, clinical trial

## Abstract

**Introduction:**

Implantable neural devices, including brain–computer interfaces and spinal cord stimulators, hold significant therapeutic promise for conditions such as paralysis and chronic pain. However, the novelty of these technologies introduces unique ethical challenges. While much of the existing literature emphasizes development-related concerns such as device safety, the ethical issues surrounding explantation remain relatively underexplored.

**Methods:**

We conducted a systematic review to identify ethical, legal, and sociocultural considerations relevant to the explantation of neural devices. The review applied the IEEE BRAIN Neuroethics framework as a guiding structure for the categorization of the themes. A subsequent thematic analysis was performed to categorize and synthesize findings across studies.

**Results:**

Thematic analysis revealed that medical motives were the predominant factor in discussions of explantation, with 83% of studies citing medical complications as a central concern. Additional themes identified included changes in cognition and behavior, emotional well-being, lack of therapeutic benefit, identity, financial issues, autonomy, post-trial considerations, and neurorights.

**Discussion:**

Our findings underscore the multifaceted nature of neural device explantation, extending beyond purely medical considerations to include psychological, financial, legal, and sociocultural dimensions. These results highlight the necessity of interdisciplinary approaches to adequately address the broad spectrum of challenges associated with explantation.

## 1 Introduction

Implantable neural devices, such as brain-computer interfaces (BCIs) and spinal cord stimulators (SCSs), have undergone significant advancements over the past decades (White et al., 2015). Developments are driven by technological progress and increased clinical effectiveness in treating conditions like epilepsy, chronic pain, and speech impairments (Grillner et al., 2016). Globally, the number of clinical trials involving implantable neurodevices has risen, with projections indicating further growth in the coming years (Harmsen et al., 2020; Zhang et al., 2020).

The novelty of neurodevices presents unique ethical challenges and, to date, research has mostly focused on development-related concerns, including trial design, safety, and informed consent (Klein, 2016; Sankary et al., 2023; Wexler et al., 2022). However, emerging issues around the explantation of neural devices are gaining attention: devices can lose functionality over time, experimental therapies might be halted due to financial constraints, and explantation itself requires surgical procedures that carry inherent risks (Higgins et al., 2024; Hansson, 2021).

To our knowledge, no comprehensive overview of the considerations regarding the explantation of neural devices exists. Such an analysis is crucial for identifying knowledge gaps, overlooked issues, and opportunities for further research, while providing actionable recommendations for future trials. Understanding the drivers of explantation is essential to maximizing patient benefit and minimizing avoidable harm.

In this article, we offer an in-depth overview of the ethical, legal, and sociocultural considerations in explantation of implantable neural devices in humans, as categorized according to the IEEE BRAIN neuroethics framework: Medical application (IEEE brain, n.d.). These considerations include categories such as safety and risk, agency and identity, authority and power, justice and fairness, and surveillance and privacy. Using this framework, we conducted a systematic review of academic literature, inspired by the methodology of Strech and Sofaer (2012), complemented with a thematic analysis. We present our findings and highlight challenges and arguments requiring attention as the field of neurodevices evolves.

## 2 Methods

This systematic review investigated considerations in explantation of neural devices in the academic literature. PRISMA (Preferred Reporting Items for Systematic Reviews and Meta-Analyses) guidelines (Page et al., 2021) were followed, with input from a librarian to optimize the search strategy. Search terms included “neural devices,” “ethics,” “explantation,” and “post-trial management,” along with their synonyms. The complete search strategy is detailed in Supplementary Data. The following databases were searched on 9 September 2023: PubMed, Embase, Web of Science, Cochrane Library, Emcare, Academic Search Premier (EBSCOhost), PsychINFO, and Philosopher’s Index (EBSCOhost). Reference lists of included studies were manually reviewed during full-text selection to identify additional relevant literature, using the snowballing method.

### 2.1 Inclusion and exclusion criteria

Included studies adhered to the following criteria: (1) focus on neural devices; (2) devices are implanted in the central nervous system and provide stimulation and/or decode electrical signals in neural tissue; (3) includes considerations of “explantation,” “removal,” “change,” or related terms; (4) if studies do not explicitly mention explantation, they must address safety and feasibility of neural devices; (5) peer-reviewed articles and comments; (6) articles in English, Dutch, French, German, or Spanish. Exclusion criteria entailed: (1) case reports or series (n ≤ 4); (2) devices implanted in the peripheral nervous system or neurodevices with a primary function to deliver drugs.

### 2.2 Selection and analysis

Four authors (MV, KB, KJ, and AB) independently screened the publications. For title and abstract screening, acceptance by two reviewers was required for inclusion, with AB resolving any disagreements. MV reviewed all studies for full-text selection, while KB and KJ each reviewed half of the studies from the initial screening. Separately, MV and KB reviewed the publications collected via the snowballing method and AB was consulted in case of disagreement. Selection was conducted independently and blinded using Rayyan software (Ouzzani et al., 2016). Extracted data were considerations related to explantation, independently extracted by MV and KB using an Excel template and categorized using the IEEE BRAIN neuroethics framework (Medical application) (IEEE brain, n.d.), which organizes themes into ethical, legal, and sociocultural domains. Subcategories of ethical considerations included:

1. Safety, Risk, and Well-being.
2. Agency and Identity.
3. Authority and Power.
4. Justice and Fairness.
5. Surveillance and Privacy.

**Figure 1:**
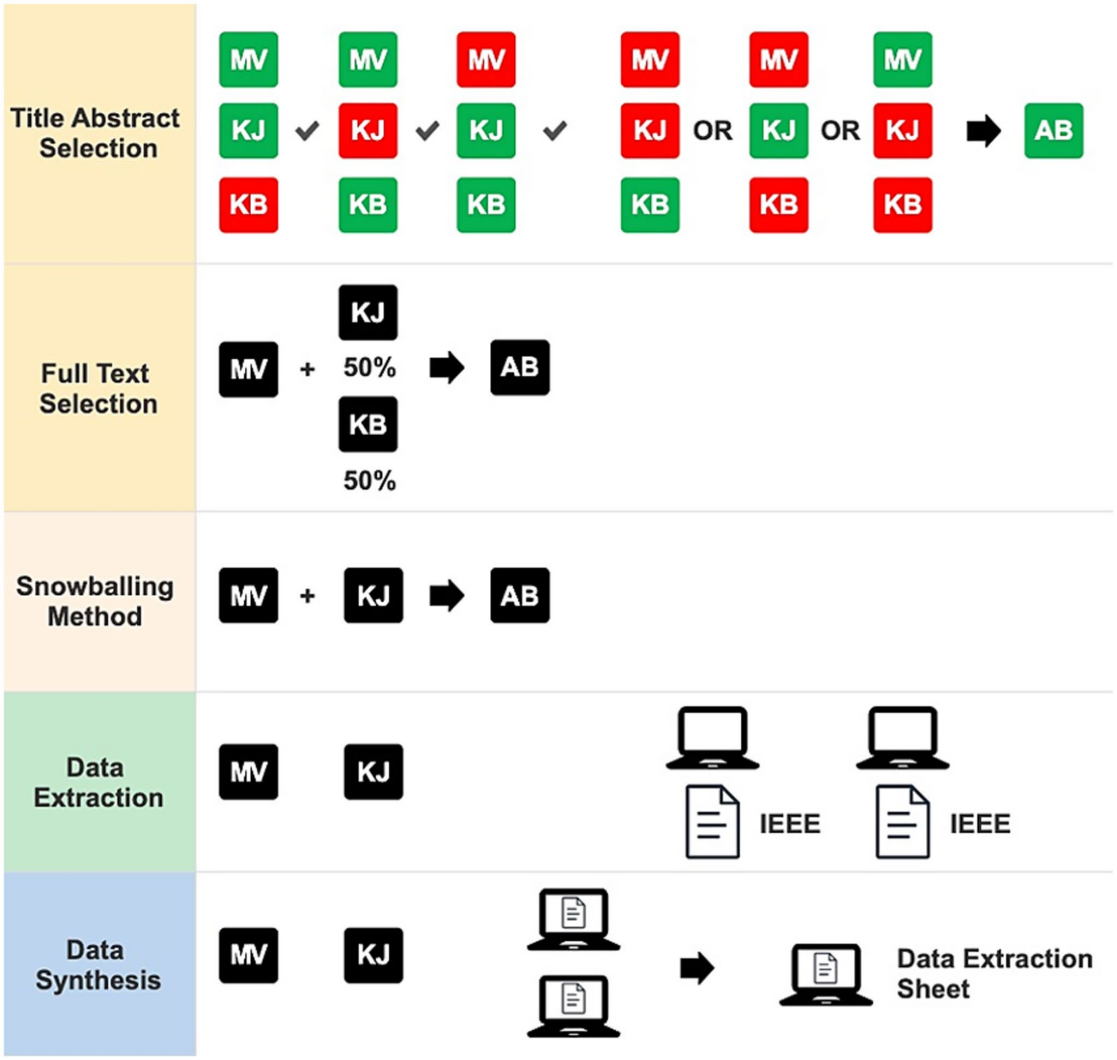
Authors contribution and data synthesis processes. MV, Manuela Vooijs; KJ, Karin Jongsma; KB, Katherine Bassil; AB, Anne Brinkman; IEEE, BRAIN neuroethics framework.

Legal considerations were further divided into Data and Regulation. Sociocultural considerations were not further subdivided. We employed the same definitions as delineated of the different themes and sub-themes in the descriptions of the IEEE Neuroethics Framework for Medical Applications. All considerations were collected and categorized based on the data extraction method described by Strech and Sofaer (2012); to minimize bias, considerations were related on the exact quotes in studies. A visual representation of author contribution in study selection and data extraction is presented in Figure 1. Furthermore, studies could be assigned to multiple subcategories of the considerations based on their content.

Additionally, general information was collected: (1) author, year, and country; (2) field of study (e.g., medical, ethical, psychological), based on the journal; (3) study type; (4) technology type (e.g., DBS); (5) study aim; (6) mention of “revision,” “reoperation,” or “change.”

## 3 Results

### 3.1 General summary

**Figure 2:**
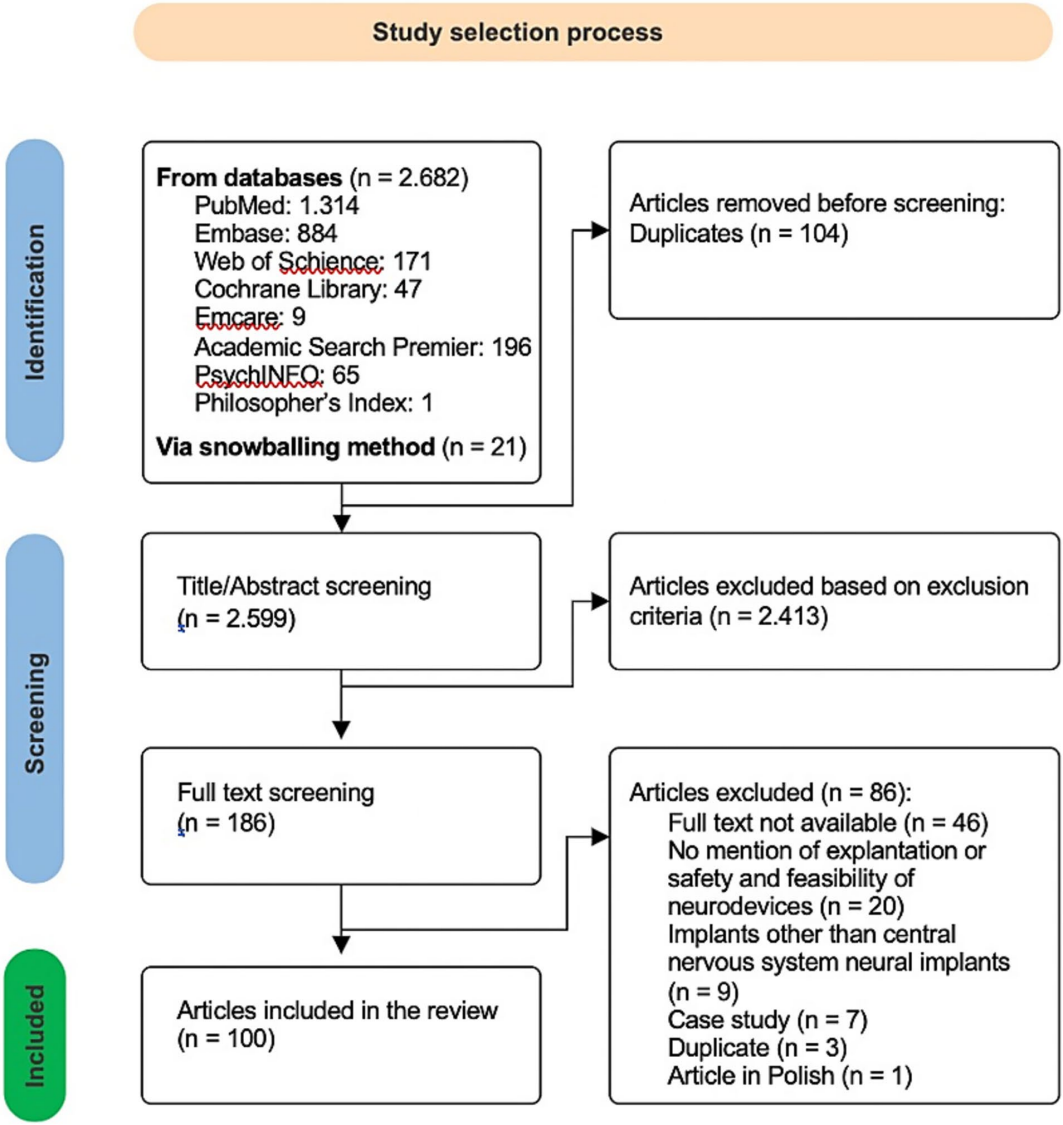
Study selection process according to the PRISMA Guidelines.

A total of 100 publications were included in this systematic review, with the study selection process summarized in the PRISMA flowchart (Figure 2). The majority of included studies were medical publications (*n* = 77), followed by ethical analyses (*n* = 20), economic evaluations (*n* = 2), and engineering studies (*n* = 1). Nearly half of the studies originated in the United States (*n* = 47).

**Figure 3:**
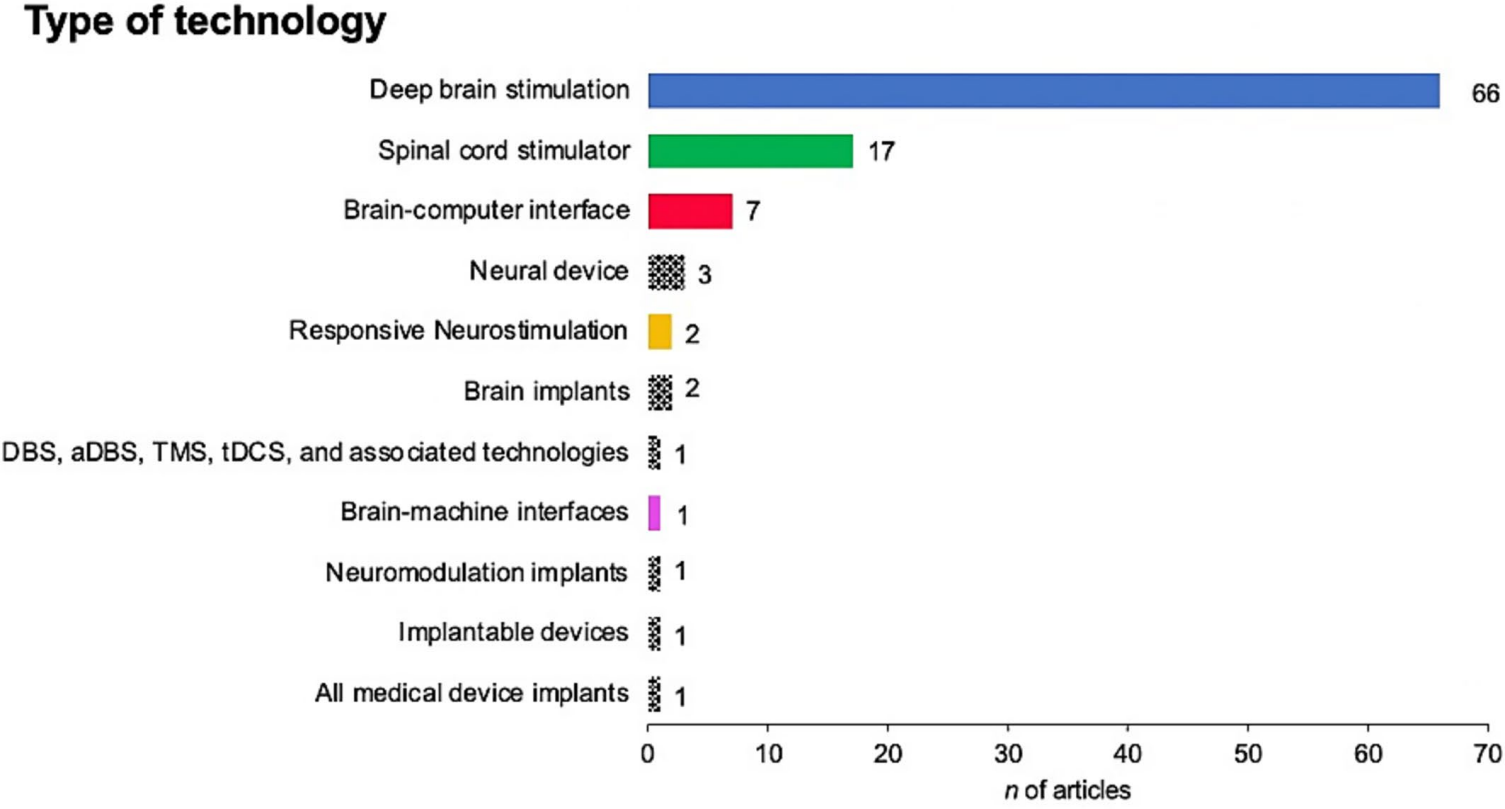
Type of technology used as mentioned in articles. DBS, Deep Brain Stimulation; aDBS, adaptive Deep Brain Stimulation; TMS, Transmagnetic Stimulation; tDCS, Transcranial Direct Current Stimulation.

**Figure 4:**
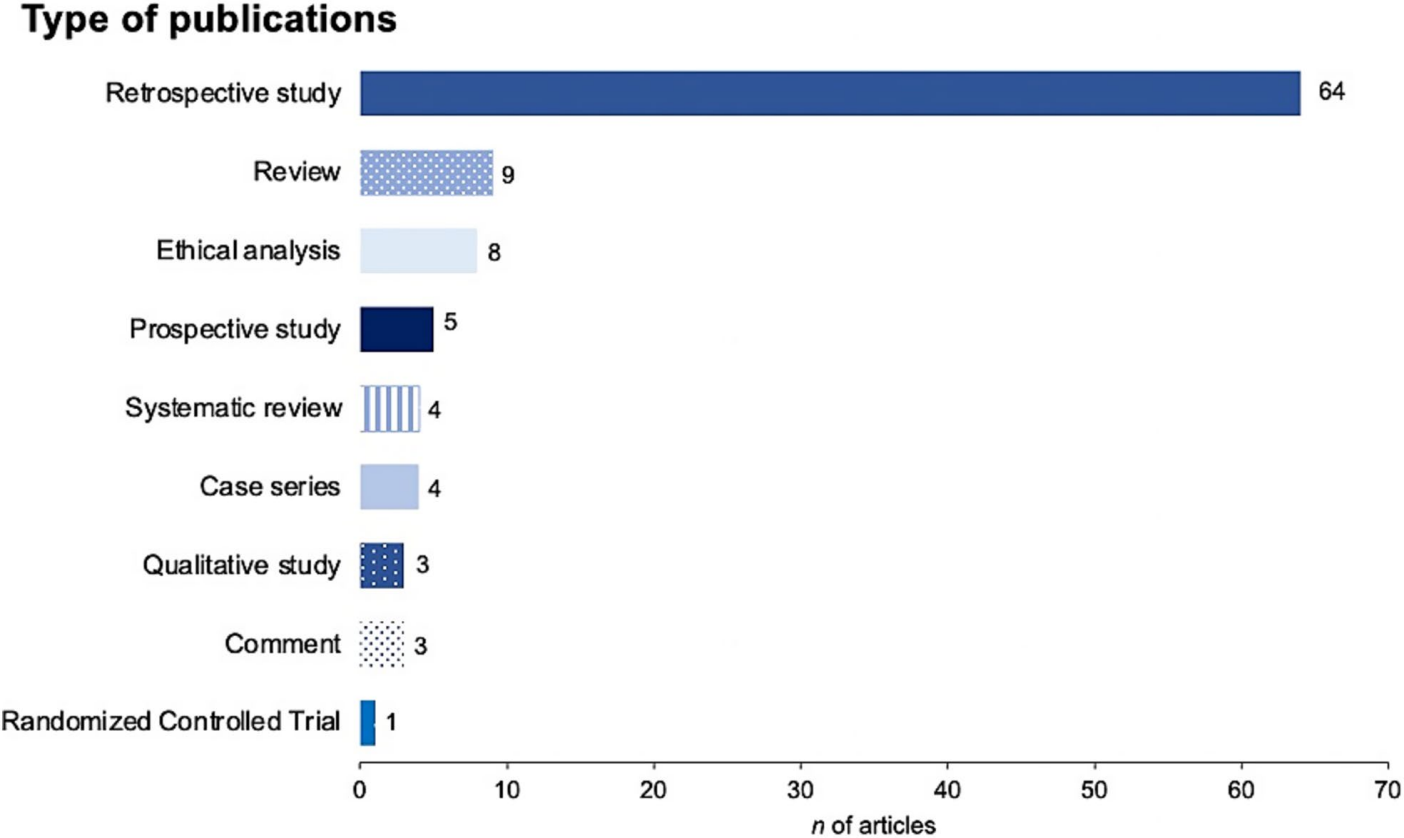
Type of publications included in final review.

Most articles concerned deep brain stimulation (DBS) systems (*n* = 66), SCS systems (*n* = 17), BCIs (*n* = 7), or neurodevices defined otherwise as shown in Figure 3. The majority of publications were retrospective studies (64%), alongside reviews, ethical analyses, and case series, as presented in Figure 4.

A general key finding was the lack of consensus on the definition of explantation. Only three studies explicitly defined explantation as the removal of neural devices (Al-Kaisy et al., 2020; Gorgulho et al., 2009; Van Buyten et al., 2017). Most studies implicitly categorized explantation based on device components, distinguishing “partial” removal (e.g., explantation of implantable pulse generators (IPGs), leads, or extensions) from “complete” removal of all components. Notably, in 44 studies, explantation did not always signify permanent device removal; instead, terms like “revision,” “battery replacement,” or “reimplantation” were used, indicating technical updates or system reinstallation (Gorgulho et al., 2009; Rajabian et al., 2022; Jakobs et al., 2023; Sobstyl et al., 2019; Pope et al., 2017; Bennett et al., 2021; Simopoulos et al., 2019; Wu et al., 2021; Kashanian et al., 2020; Wirth et al., 2021; Frizon et al., 2018; Engel et al., 2018; Visser et al., 2021; Powers et al., 2023; Grimaldi et al., 2023; Spindler et al., 2023; Bouwens Van Der Vlis et al., 2022; Wakim et al., 2021; Narváez-Martínez et al., 2019; Reuter et al., 2018; Kim et al., 2017; Fernández-Pajarín et al., 2017; Chen et al., 2017; Kaminska et al., 2017; Ponce et al., 2016; Hayek et al., 2015; Pepper et al., 2013; Piacentino et al., 2011; Fily et al., 2011; Servello et al., 2011; Vergani et al., 2010; Bhatia et al., 2010; Voges, 2006; Lyons et al., 2004; Temel et al., 2004; Oh et al., 2002; Rauck et al., 2023; De La Porte and Van De Keift, 1993; Kiss and Becker, 2012; Fytagoridis et al., 2016; Hendriks et al., 2019; Lázaro-Muñoz et al., 2018; Bullard et al., 2020).

Additional general information about the included studies is detailed in Supplementary Data Sheet 1 and an overview of considerations is provided in Supplementary Data Sheet 2.

### 3.2 Ethical considerations

#### 3.2.1 Safety, risk and well-being

The primary considerations for explantation revolved around safety, risk, and well-being. In decision-making revolving around neurodevice removal, stakeholders weigh the potential harms and benefits to users reflecting the bioethical principles of *non-maleficence* and *beneficence*, respectively.

Medical complications were in fact the most reported consideration in explantation. In total, 83% of the studies reported partial or complete removal of the neurodevice as a consequence of medical complications, ranging from infection to neurological impairments. Infection was most frequently mentioned as a reason for device removal (*n* = 67). Reported rates of infection ranged from 1.6% (Fernández-Pajarín et al., 2017) to 46.7% (Deeb et al., 2020) and consisted of surgical site infections (SSIs) or deeper infections such as meningitis. These 67 studies also differed in their medical approach to salvage the neurodevice, reflecting different harm-versus-benefit analyses. On one side of this balance, the decision to explant the neural device was based on the severity of infection, with infiltration of the infection to the intracerebral space as an important parameter for potential *harm* to the patient (Bazaka and Jacob, 2012). On the other side of the balance, that of *beneficence*, studies mostly mentioned salvage protocols for infection in order to rescue the device. Six studies described how importance was given to this *therapeutic benefit* of the neurodevice in the case of superficial infections, by first attempting antibiotic treatment and explanting the device only when longer durations of antibiotics were unsuccessful (Kashanian et al., 2020; Spindler et al., 2023; Bhatia et al., 2010; Bullard et al., 2020; Jitkritsadakul et al., 2017; Wetzelaer et al., 2018). Few studies described the in-depth decision-making process regarding explantation for infection (Bhatia et al., 2010; Bullard et al., 2020; Hagedorn et al., 2021). Hagedorn et al. (2021) for example, enumerated the factors involved in their infection protocol: the decision to explant was made case by case and was based on the degree of infection, clinical response, and the dependence of the patient on the DBS system.

Other medical complications that prompted device removal were hydrocephalus, hemorrhage, cerebrospinal fluid (CSF) leak, and psychiatric symptoms and disorders such as depersonalization and suicide. Another medical complication was reported as a reason *against* explantation by several studies. Withdrawal syndrome, following explantation of DBS systems in Parkinson’s disease (Grimaldi et al., 2023; Reuter et al., 2018; Chen et al., 2017; Kaminska et al., 2017; Jitkritsadakul et al., 2017; Hariz, 2016), was described in a few patients who experienced severe parkinsonian crises, including dysautonomia—a state in which the major organ systems and processes such as heart rate and blood pressure are dysregulated—leading to severe medical emergencies and near-death situations in intensive care settings (Grimaldi et al., 2023; Reuter et al., 2018; Chen et al., 2017).

Following medical complications, the second most reported consideration in explantation was “hardware” complications. In total, 44 studies (44%) reported hardware malfunction as a reason for explantation or revision of neural implants, including extensor cable breakage, lead migration or fracture, tethering of lead extensions, sudden battery failure, IPG dislocation, and high impedance (Al-Kaisy et al., 2020; Pope et al., 2017; Wirth et al., 2021; Kaminska et al., 2017; Pepper et al., 2013; Vergani et al., 2010; Lyons et al., 2004; Oh et al., 2002; De La Porte and Van De Keift, 1993; Hariz, 2016; Sillay et al., 2008).

In comparison to medical and technical complications, shifts in cognition and behavior were described in only a few studies. Changes in cognition and behavior can be attributed to changes in neural networks, impacting cognitive abilities such as memory, attention, and executive function. In total, 6 studies reported explantation of neural implants based on these cognitive and behavioral alterations (Rajabian et al., 2022; Tabaja et al., 2023; Gilbert et al., 2023; Gilbert, 2013; Clausen, 2009). Transient behavioral changes were reported in 4.2% of DBS procedures in a trial that investigated the efficacy of intraoperative MRI, mostly including confusion (2.5%), low mood (1.1%), and suicidal ideation—of which one patient experienced self-harming behavior (0.6%) (Rajabian et al., 2022). Moreover, two qualitative studies focused on the experiences of patients. The first trial consisted of DBS for depression, in which one participant described explantation as removal of the treatment that she felt was keeping her alive (Tabaja et al., 2023). Neural implants becoming an integral part of a person also arose in the other trial, in which patients received experimental implantable devices that predict seizures and give neurostimulation to prevent the suspected insult. Gilbert et al. (2023) reported the experience of a patient who experienced confusion, disorientation, and anxiety following explantation of the neural implant. The authors stated that the neurodevice had influenced her cognitive and emotional processes, and, following from these subjective experiences, they raised the concern that explantation of neural devices may violate one’s right to “psychological continuity” and “mental integrity” (Gilbert et al., 2023). In another ethical analysis, Gilbert (2013) repeated the argument that neural devices may change the “self” and promote a sense of powerlessness or loss of control. In one study, the impact of neural implants on behavior brought about a complex scenario, in which a woman with obsessive-compulsive disorder (OCD) had no *therapeutic benefit* from neurostimulation but experienced an evident increase in happiness (Clausen, 2009). She asked for continued stimulation, despite a lack of benefit from the therapy. Ultimately, the study did not enclose the final decision regarding explantation of the neurostimulator, but it described the complexities in the definition of *benefit*.

While these studies described patients’ cognition and behavior—in simpler terms, their thinking and doing—some also touched upon emotional well-being. These three concepts are inherently interconnected, and emotional well-being here is defined as one’s experience of emotions and processing of these internal sensations. Emotional well-being was explicitly addressed by Gilbert et al. (2023), who described altered emotional states after BCI explantation, and by Gilbert (2013), who noted a potential sense of powerlessness following explantation. Additionally, two studies further explored emotional well-being in research settings. The first study, the BrainAble project, examined BCI applications for individuals with complex disabilities such as amyotrophic lateral sclerosis (ALS) and motor neuron disease (MND) and emphasized emotional well-being as a key ethical consideration in their research design (Carmichael and Carmichael, 2014). The researchers recognized the vulnerability of participants with these progressive, terminal conditions leading to severe neurological impairments such as locked-in syndrome and emphasized the importance of sensitive communication about BCIs—avoiding language that might inadvertently draw attention to the dire prognosis. Additionally, they developed an ethics framework that reflects participants’ emotional challenges, acknowledging the impact of emotional lability that may be associated with their medical conditions. Secondly, Sankary et al. (2023) investigated emotional well-being among explanted participants, noting unique challenges in this context. Overall, these studies are unique in their focus on emotional well-being in the neurodevice literature.

Of crucial note is that thorough psychological and psychiatric assessment before implantation was lacking in the aforementioned studies—apart from the case report in which *n* = 1, where the patient had OCD (Clausen, 2009)—which makes it difficult to generalize results or state inferences between neural implants and psychological complaints. These findings merely suggest that subtle forms of “psychological harm” may be present in cases of explantation. Two studies that did investigate the relationship between explantation and psychiatric comorbidities, focused on SCS only. Both studies found increased explantation rates of spinal cord stimulators in patients with psychiatric comorbidities. Beletsky et al. (2023) reported explantation rates of 14.7% versus 8.8% (*p* < 0.001) in the psychiatric versus non-psychiatric group. Patients with five psychiatric disorders before implantation had greater odds of explantation compared to the non-psychiatric group, translating to an odds ratio of 4.25 (95% CI = 1.121–16.11, *p* = 0.03). Patel et al. (2018) found a similar trend in patients with post-traumatic stress disorder and depression or anxiety. Patients with such diagnoses were more likely to undergo explantation before 1 year. Although the data are slim, these findings suggest an important role for cognition, behavior, and emotional well-being in decisions regarding explantation. Since these studies only regard SCS, they cannot be extrapolated to other neural implants such as responsive neurostimulation and DBS.

While aforementioned considerations mostly revolve around *harm*, there were also studies that looked at the *benefit* arm of the analysis in explantation. Several studies implicitly focused on *therapeutic benefit* with regards to explantation. The interpretation of *therapeutic benefit*, however, often remained unclear and could range from measurable outcomes to subjective patient-reported benefits. On the opposite side of the spectrum, *a lack of benefit*, was reported by 26 studies as a reason to explant the neurodevice, researching SCS (*n* = 12 of all studies, 46.2%), DBS (*n* = 12, 46.2%), RNS (responsive neurostimulation) (*n* = 1, 3.8%), and neural devices as a whole (*n* = 1, 3.8%) (Al-Kaisy et al., 2020; Van Buyten et al., 2017; Pope et al., 2017; Bennett et al., 2021; Simopoulos et al., 2019; Frizon et al., 2018; Powers et al., 2023; Bouwens Van Der Vlis et al., 2022; Wakim et al., 2021; Kim et al., 2017; Hayek et al., 2015; Lyons et al., 2004; Oh et al., 2002; Rauck et al., 2023; De La Porte and Van De Keift, 1993; Kiss and Becker, 2012; Deeb et al., 2020; Hagedorn et al., 2021; Patel et al., 2018; Gilbert et al., 2022; Maldonado-Naranjo et al., 2018; Wang et al., 2021; Olaciregui Dague et al., 2023; Zawar et al., 2021; Liu et al., 2013; Dupré et al., 2018). The underlying causes for inefficacy or decreased benefit of neural implants varied per disease and type of neurodevice. Lack of benefit was at times due to effective treatment of the neural implant up to the point of symptom resolution. For Tourette syndrome, this “*lack of benefit”* was described as resolution of tics and led to subsequent explantation of the DBS (Kimura et al., 2021). In the case of pain, 5 studies described complete resolution or remission of pain as a reason to remove the SCS (Van Buyten et al., 2017; Simopoulos et al., 2019; Hagedorn et al., 2021; Patel et al., 2018; Dupré et al., 2018). For the remaining studies (*n* = 26, 26%) that reported on lack or loss of benefit, the underlying reason for this inefficacy of the neural implant is unclear (Al-Kaisy et al., 2020; Van Buyten et al., 2017; Pope et al., 2017; Bennett et al., 2021; Simopoulos et al., 2019; Frizon et al., 2018; Powers et al., 2023; Bouwens Van Der Vlis et al., 2022; Wakim et al., 2021; Kim et al., 2017; Hayek et al., 2015; Lyons et al., 2004; Kiss and Becker, 2012; Deeb et al., 2020; Hagedorn et al., 2021; Patel et al., 2018; Gilbert et al., 2022; Maldonado-Naranjo et al., 2018; Wang et al., 2021; Olaciregui Dague et al., 2023; Zawar et al., 2021; Liu et al., 2013; Dupré et al., 2018).

#### 3.2.2 Agency and identity

Agency and identity play a crucial role in the clinical and research setting. In the clinic, patients must be included in the decision-making regarding neurodevices by the medical principle of autonomy. In trials, participants must be able to make informed decisions and decide on trial participation or exit without coercion.

Several studies present situations in which neural implants are removed for reasons pertaining to agency and identity. The principle of autonomy in the considerations on explantation is reflected either explicitly or implicitly in twelve studies (12%) (Hansson, 2021; Hayek et al., 2015; Hendriks et al., 2019; Gilbert et al., 2023; Gilbert et al., 2022; Zawar et al., 2021; Sierra-Mercado et al., 2019; Zuk et al., 2018; Gilbert, 2015; Kaptan et al., 2020; Ginalis et al., 2021; Klein and Ojemann, 2016).

A few studies (*n* = 7) mentioned *identity* as an integral part of their discussion on explantation of neurodevices. Clausen (2009), Gilbert et al. (2023), Gilbert (2013), and Sankary et al. (2023) noted post-operative changes to the patient’s identity, such as feelings of becoming a “machine,” powerlessness, loss of control, but also an increased capacity due to the technology, resulting in a sense of liberty. Klein (2016) reported the potential effects of DBS on personality, such as a loss of interest in a patient’s preoccupation which was difficult to distinguish and/or eventually led to psychiatric disorders, such as mania.

Reflecting the principle of autonomy, certain authors report on the patient’s desire for removal as a reason for explantation (Hayek et al., 2015; Zawar et al., 2021). Such situations give rise to a primary focus on autonomy, which is described by some authors as problematic. Hansson (2021) outlines the scenario in which patients’ survival relies heavily on the neurodevice. Explantation, in those cases, is synonymous with the death of the patient—for example, the explantation of DBS in Parkinson’s disease resulting in severe parkinsonian crises. Respecting their decision for removal thus may lead to severe disease burden, asking for complex medical decisions and causing tension with the principle of *non-maleficence* (Hansson, 2021).

Another counterargument for explantation based on the patient’s autonomy in the literature was a reduced capacity for decision-making during a trial or therapy. In other words, patients might not be able to make the decision in their own best interest, perhaps due to disease progression or the introduction of the neural implant itself. In these cases, this capacity influences the decision for explantation, and patients might become more dependent on researchers or treating physicians (Klein, 2016). A complex real-life example was illustrated by Gilbert (2013), in which a DBS-related device was used as experimental therapy for treatment-resistant depression. After implantation, the patient attempted suicide by overdose and reported feelings of “depersonalization” that were not present pre-implantation. The medical team then advocated for explantation due to lack of benefit and a possible association with suicidal ideations (Clausen, 2009).

Several studies raise a different concern: “medical indications” motivating explantation inherently have a degree of coerciveness, as patients may not feel able to influence this decision for explantation. Some studies labeled this phenomenon “forced explantation” (Hansson, 2021; Clausen, 2009; Gilbert et al., 2022; Zuk et al., 2018). As described by Gilbert, in these cases, the concept of “bodily integrity” is frequently neglected. He states: “Performing explantation resulting from some degrees of coerciveness may place patients at greater risk of harms, and often for no apparent benefit other than removing the device.” (Clausen, 2009).

#### 3.2.3 Authority and power

Authority and power in medical and research settings are critical concepts that shape the relationships between professionals and patients or participants. While authority refers to the legitimate ability of medical and research teams to make decisions, power involves the ability to enforce those decisions.

Gilbert et al. (2022) raise the subject of bodily integrity and non-consensual explantation. They describe that explantation requires adequate informed consent. The difficulty here is that informed consent regarding implantation does not equate informed consent regarding explantation. These two moments should be assessed separately. In a real-world example, this means that patients who are implanted after informed consent procedures, in which the risk for infection is also mentioned, are still able to refuse explantation in the case of infection. In Gilbert et al. (2022) words, explantation on medical grounds would be equivalent to using coercive power of medical professionals to override patient’s right to bodily integrity.

The complexity of informed consent in neural implant research becomes evident in research by Sankary et al. (2023). In semi-structured interviews, participants from DBS and RNS trials were interviewed on their reason for joining and exiting or withdrawing from trials to understand exit processes and participant’s decision-making in this phase. Participants were often hoping for symptom relief. This motive to join the trial left them blinded for future long-term implications of research participation. One patient noted that he “[…] did not really focus on the end except [for] hoping to have some relief” (Sankary et al., 2023). This phenomenon—the blurring of research and care—is known as “therapeutic misconception” and can become problematic when the research design, including descriptions of when neural implants will be removed, is not in line with participants’ expectations. Klein and Ojemann (2016) point at another example that research participants often do not foresee upon trial participation. Neural implants sometimes require extensive training for optimal functioning. The inability to recognize the effort, time, and resources may lead to premature exit from research—and thus, explantation before the neurodevice can offer any therapeutic benefit (Klein and Ojemann, 2016).

While these studies mostly focus on patients in the power dynamics between patients and medical and research authority, Hansson (2021) shines light on the side of the physician. He describes that patients may wish for removal of neural implants, even when death inevitably follows explantation—a scenario which Hansson (2021) does not further illustrate and did not appear elsewhere in the literature but might resemble cases of fatal withdrawal syndrome following DBS explantation. Helping a patient to die, by removing the neurodevice, is a criminal offence in many countries. Besides this legal issue, this scenario highlights the ethical responsibility of physicians in end-of-life decisions. Physicians must balance their duty to respect patient autonomy with the ethical and legal implications of their actions, ensuring that their interventions align with both professional standards and the protection of life.

#### 3.2.4 Justice and fairness

Considerations regarding justice and fairness primarily entail the ethical obligations of researchers and medical institutions to address participants’ needs. Studies report on financial burdens and post-trial considerations such as continued access to care.

Several studies highlighted the financial responsibilities associated with devices used in medical research, particularly regarding explantation costs, device maintenance, and the burden placed on participants when the costs are not covered by sponsors or insurance. For example, Gilbert et al. (2023) described a case where patients were forced to undergo explantation due to financial constraints of the neurodevice trial. Participants were willing to place high financial burdens on themselves, such as taking second mortgages, if it meant for them to keep the device. Contrastingly, Sierra-Mercado et al. (2019) offered a scenario in which the principle of justice is endangered at the end of trial participation when patients do not benefit from the device. Explantation, in this scenario, may have to be covered by participants themselves—especially as insurance companies do not cover costs associated with investigational implants (Hendriks et al., 2019).

The second theme that arose from this review was post-trial considerations, as shortly described above. Different scenarios for neurodevices are possible after trial end, the device is (1) inactivated but remains implanted, (2) is active with continued care and follow-up, and (3) is explanted (Sierra-Mercado et al., 2019). Several studies looked at the duties of researchers with regard to trial end, exits and continued access to the neurodevices (Sankary et al., 2023; Hansson, 2021; Lázaro-Muñoz et al., 2018; Gilbert et al., 2023; Sierra-Mercado et al., 2019). Reasons for explantation at trial end vary and as mentioned earlier may be fueled by financial motives. Another reason for explantation, proposed by Gilbert et al. (2022), is that explantation is deemed ethical by review boards, since the therapeutic benefit of the implant is not yet known.

The obligation of researchers to continue access to neurodevices is discussed in several publications (Sankary et al., 2023; Hansson, 2021; Pope et al., 2017; Hendriks et al., 2019; Lázaro-Muñoz et al., 2018; Gilbert et al., 2023; Clausen, 2009; Carmichael and Carmichael, 2014; Gilbert et al., 2022; Sierra-Mercado et al., 2019; Zuk et al., 2018). Multiple authors recognize the vulnerability of participants in neurodevice research as they lend their bodies to science and undergo invasive procedures that place burden on participants (Hendriks et al., 2019; Lázaro-Muñoz et al., 2018; Gilbert et al., 2022; Sierra-Mercado et al., 2019; Zuk et al., 2018).

Sierra-Mercado et al. (2019) argue that this burden should be shared between participants and researchers. By the duty of non-abandonment, researchers should provide continued access to care and, therefore, a reason against explantation by default per study design or because of financial constraints (Hansson, 2021). From another perspective, non-abandonment might also imply explantation when participants wish to return to their pre-trial state. In this case, researchers should facilitate the preference for removal, including costs associated with explantation (Sierra-Mercado et al., 2019). Sierra-Mercado et al. (2019) and Zuk et al. (2018) propose a conflict that arises from these post-trial responsibilities: the goal of research is to develop interventions that benefit the patient population. Simultaneously, the extent to which researchers have to acknowledge participants’ wish for continued access post-trial may not be compatible with the sustainability of research in general. In other words, if post-trial access means lifelong care, updates, and access to neural implants—with associated costs and expertise—this might hinder researchers from realizing these studies in the first place (Sierra-Mercado et al., 2019). Along these lines, Sierra-Mercado et al. (2019) state that researchers should not be obligated to cover costs of explantation in a case of a participant’s preference if this is not compatible with sustainability of the research. Put differently, if studies end and explantation is not necessarily required, this perspective might mean that patients pay the costs of explantation themselves. Contrastingly, Hansson (2021) argues that researchers must provide access to participants who benefit from neural implants after the research. In all, the duty of non-abandonment implies responsibilities of researchers to care for patients while also maintaining the continuity of the research itself. The relevance of clear post-trial management is illustrated by the fact that, based on the current literature, early clinical brain implant studies might end in explantation (Sankary et al., 2023; Pope et al., 2017; Gilbert et al., 2022).

#### 3.2.5 Surveillance and privacy

Surveillance and privacy are the ethical subcategories that shine light on neural implant data storage, collection, usage, and analysis. These themes were not mentioned in the literature regarding explantation of neurodevices.

### 3.3 Legal considerations

Legal considerations for or against explantation can be divided into two subcategories: Data and Regulation—as stated by the IEEE Neuroethics framework (IEEE brain, n.d.).

#### 3.3.1 Data

Legal considerations in relation to data vary depends on whether the use of neural data is personal or by third parties. This includes all forms of neural data derived, analysed, or extracted from neural implants as well as different ways in which data is manipulated. It also includes ownership of data and consent for sharing or abstaining from sharing data with third parties. No study specifically addressed data-related legal considerations in relation to explantation.

#### 3.3.2 Regulation

Regulatory concerns surrounding medical neurotechnology include the complexity and variability of regulatory regimes across jurisdictions, particularly with respect to how medical neurotechnologies are classified and the degree of oversight deemed necessary based on their invasiveness and potential for harm. Across the studies reviewed, there is a growing recognition that existing legal frameworks may be inadequate to address the unique explantation risks these devices pose—not only to physical integrity but also to cognitive functions and personal identity.

Overall, two studies listed legal reasons for explantation; both studies were ethical analyses. Gilbert et al. (2023) raised the concern of the explanted patient as a new legal personhood. They drew from the example of “patient R,” a participant to a Neurovista trial for chronic refractory epilepsy whose life drastically improved after receiving a BCI implant to control seizures. She stated to feel synergistic with the device—“it was me,” “we became one”—and was able to do more now that the seizures were under control. Based on this renewed sense of self and a gain in ability, the authors stated that patients might be granted legal protection based on the fact that they are now a “postoperative symbiotic person”—in symbiosis with the neurodevice. Therefore, the post-operative person should be asked and consent for explantation. In other words, consent for implantation is not a synonym for consent for explantation, and therefore consent should be collected at least twice: before implantation, from the preoperative person, and postoperative, from the “symbiotic person.”

Gilbert et al. (2023), refer to these rights as “neurorights.” These might prevent explantation of investigational neural implants when the sponsor does not have the capacity for continued support or when the trial is ended for financial reasons. In the case of patient R, the Neurovista trial was ended due to financial issues and continued access to the device, including technical updates and care, was not financially feasible. Patient R was reluctant to explant the device and even considered a second mortgage to keep the implant. Ultimately, she consented to explantation which was not without emotional consequences: “To finally switch off my device was the beginning of a mourning period for me […].” Neurorights were proposed by the authors as a means of protection for participants by viewing implanted patients as new legal personhoods that require additional consent. Such rights aid in the creation of new laws that offer legal borders in the definition of implanted and explanted patients and allow for safe, ethical research (Gilbert et al., 2023).

Legal considerations extend further than the rights of the patient or user. A reason for explantation, as mentioned by Hansson (2021), is recall of the device by the manufacturer. This might happen in cases of software malfunction or errors in production that have arisen after implementation. In these cases, Hansson (2021) discusses that the call by the manufacturer should not automatically indicate explantation, especially when removal involves risky procedures for the patient. Currently, this is an ethical and legal grey zone which calls for legal device regulation systems that guide manufacturers, physicians, and patients in this process and informs them about their duties and rights.

### 3.4 Sociocultural considerations

Sociocultural factors play a substantial role in the decision-making process regarding the explantation of neural implants. This review examines these factors from both the individual patient’s perspective and broader societal and healthcare-driven considerations. No specific cultural considerations were reported in any of the studies.

First, individual patient factors such as social relationships, perceptions of physical appearance, and financial status are critical in decisions to either retain or remove a neural implant. The social context of a neural implant user—including relationships with family, friends, and, in research settings, with the research team—can strongly influence the decision to undergo explantation. Clausen (2009) cited a French study in which Parkinson’s patients, despite experiencing improved neurological function from neurostimulation, faced challenges in psychosocial adjustments, particularly in their professional and familial relationships. In another study, Sankary et al. (2023) emphasized the role of the relationship between participants and researchers, suggesting that strong rapport with the research team can positively influence participants’ ability to cope with the outcomes of the study. While not directly influencing explantation decisions, these findings underscore how social relationships can shape an individual’s experience and perception of neural implants in a research context.

In addition, concerns regarding the physical appearance of neural implants have been identified as a factor influencing explantation decisions. Two studies specifically noted cosmetic reasons as a motivator for explantation. In one study, three patients requested repositioning of the implanted pulse generator for aesthetic reasons (Hansson, 2021), which was classified by the authors as “IPG complications” (Ponce et al., 2016). Although the exact nature of the cosmetic complaint remains unclear, this highlights the need for discussions with patients about potential scarring or other physical changes as a result of implantation.

Financial considerations also emerge as an important factor in explantation decisions, particularly in commercially available implants that require out-of-pocket payments by patients. Literature on financial motivations is limited, but the small group of studies that do exist highlight the burden associated with maintaining or removing neurodevices. First of all, Hariz (2016) presented two patients who developed a parkinsonian crisis after they were unable to afford necessary emergency replacement of their DBS. Secondly, a qualitative study by Gilbert et al. (2023) demonstrated the social impact following explantation as a result of financial constraints in the Neurovista trial. One participant—patient R—expressed deep emotional commitment to retaining the device and her husband reported to investigate taking a second mortgage to keep the device. Examples like these are not unique—as mentioned earlier under ethical considerations, *3.2.4 Justice and fairness*—and impact not only the individual, but their social environment too.

Aside from the patient-centered perspective, explantation decision-making also involves broader considerations spanning healthcare institutions’ logistics and finances. Neurodevice removal requires skilled surgical teams, operating theaters, advanced intraoperative imaging, and anesthesiologists, all of which incur substantial costs. Additionally, preoperative and postoperative care adds to the overall sum. Few studies have investigated the specific costs associated with explantation. For instance, Han et al. (2017) examined health resource utilization following SCS explantation and found that explanted patients incurred significantly higher treatment costs, with a median of $42,140.30 compared to $27,821.70 for non-explant patients (*p* < 0.0001). Moreover, the explant cohort had 3.62 times more hospital admissions than the non-explant cohort [95% CI (1.42, 9.26), *p* = 0.007]. Chen et al. (2017) reported the mean cost of a single DBS explantation to be $12,729 ± 3,284, with Wetzelaer et al. (2018) finding costs of €29,301 for IPG explantation and €9,499 for salvage procedures.

## 4 Discussion

To our knowledge, this review represents the first systematic examination of considerations surrounding the explantation of neural devices as reported in the academic literature. The findings reveal a diverse array of reasons underpinning explantation decisions, providing a comprehensive descriptive overview that serves as a foundation for future empirical research and normative ethical analysis. This systematic review encompasses 100 publications, predominantly from medical literature (77%), followed by ethical (20%), economic (2%), and engineering (1%) studies.

### 4.1 Main findings and the current literature

#### 4.1.1 Defining explantation

One of our main findings is the substantial variability in the definition of explantation across the literature. While some studies describe it in terms of partial and complete hardware, others resort to explantation without description. In their systematic review of adverse events (AEs) in DBS, Engel et al. (2018) similarly emphasized this variation and pointed at a need for clear categories to register complications. Their proposed classification—including the categories “intracranial AEs,” “hardware removal due to,” and “lead revision or indication for lead revision due to.”—offers an illustrative approach and allows for comparison between studies. The downside to this categorization is that it primarily addresses neurosurgical complications such as brain abscesses and intracranial hemorrhage. This focus risks underrepresenting psychiatric complications such as mania, which are often misclassified under broader labels like “no or suboptimal clinical effect.” An international validated definition for “explantation” and a comprehensive framework that integrates surgical, psychiatric, and psychosocial complications are therefore crucial as they allow for consistent comparison of AEs across institutions. Ultimately, this aids in assessing risk versus benefit across neurodevices and patient populations, ensuring safety and efficacy in research and experimental therapy applications.

#### 4.1.2 Motives for explantation

This review highlights a predominant focus on medical professionals’ perspectives in decisions to explant neurodevices, with limited attention to user experiences. The most frequently cited reasons for removal were complications, which ranged from medical complications such as hemorrhage to hardware-related problems such as increased lead impedance. Notably, 83% of the included studies identified complications as a reason for explantation of neurodevices. Remarkably, the perspectives and considerations of device users were rarely reported. While some studies noted “lack of therapeutic benefit” or “resolution of symptoms” as reasons for explantation they rarely explored underlying causes or broader patient outcomes. This is in line with findings of a recent qualitative review on the subjective experience of neurotechnology users (Starke et al., 2024). This study revealed a relative focus on technical aspects and underrepresentation of the user’s identity, agency, and emotions in the literature. Similarly, in our review, some studies acknowledged patient outcomes by citing “lack of therapeutic benefit” as a reason for removal, but they rarely explored the underlying causes of this lack of benefit. Moreover, “resolution of symptoms” was a recurring theme for explantation in both SCS and DBS. However, the distinct pathophysiology of conditions treated by these devices makes it unclear to what extent these reasons are comparable. For instance, in the whole cohort of neural devices reports, relatively more SCS (*n* = 12, whole cohort = 17, 70.6%) than DBS (*n* = 12, whole cohort = 66, 18.2%) reported explantation as a consequence of a lack or loss of benefit. This raises important questions: How is symptom resolution measured? How long should patient remain symptom-free to justify device removal? Are psychological and social factors considered when assessing resolution of symptoms or lack of benefit?

#### 4.1.3 Psychological harm

From the category *safety, risk and well-being*, a complex interplay between cognition, behavior, and well-being emerges from the literature. Overall, changes in cognition and behavior were reasons for explantation in 6 studies (6%) and ranged from confusion to self-harming behavior.

Explantation itself also had implications on patients’ cognition and emotion: a patient with a neural implant for seizure therapy, for example, experienced confusion, disorientation, and anxiety after device removal. Gilbert et al. (2023) used the term “psychological continuity” to describe a stable sense of self—being in charge of one’s cognition, emotion, and behavior—and advocated for users’ right in neural implant therapies. Although they do not explicitly use this term, several studies report changes in one’s identity after implantation ranging from feeling “like a machine” to gaining new abilities due to effective therapy (Gilbert et al., 2023; Clausen, 2009). For explantation, some studies reported disruptions to psychological continuity; patients experienced “depersonalization” or even changes in identity and self-perception (Clausen, 2009). Ideally, researchers and physicians would assess the psychological status of patients before and after implantation and explantation to observe changes within individuals. Assessing psychological continuity is inherently challenging, as it lacks standardized criteria like the DSM-V guidelines available for diagnosing of symptoms of psychiatric conditions like mania or depression. Similarly, this review yielded a limited number of studies that include psychological assessments before and after explantation. Although certain studies mentioned psychological assessments as part of their patient selection process; none reported systematic evaluations of psychological or psychiatric outcomes following implantation or before and after explantation. Comprehensive assessment of psychological status seems crucial, especially after the knowledge that explantation rates are higher in patient groups with psychiatric comorbidities. In doing so, researchers and physicians are able to gain better insight in potential domains of psychological harm in neural implant users.

#### 4.1.4 Responsibilities in the face of death

This review also found suicide as a potential “complication” of DBS devices and, in some cases, a driving factor in explantation. The case of a woman with treatment-resistant depression (TRD) who, despite multiple suicide attempts linked to DBS treatment, refused explantation reflects the ethical dilemmas on neural implants and death. In this case, the decision to not remove the device—adhering to the patient’s wish—may result in a successful suicide attempt and raises questions on the ethical responsibility of physicians. Hansson (2021) described this responsibility by offering a different example, namely, that of patients wishing for device removal—even when death inevitably follows explantation. In the case of neural implants, this might translate to patient’s with Parkinson’s disease who face a risk of developing withdrawal syndrome and subsequent severe medical emergencies. These studies show that explantation of neural implants in current or future life-threatening situations appeals to the ethical responsibility of physicians regarding patients’ mortality.

#### 4.1.5 Autonomy and justice

Beyond the predominant focus on risk and, to a lesser extent, beneficence in the majority of papers, considerations of autonomy and justice were less frequently reported. This imbalance may be explained by the fact that most papers were published in medical journals, where medical risks and side effects often overshadow patient-centered ethical principles in decision-making. Nevertheless, autonomy and justice are highly relevant when discussing explantation of neural implants. Depending on the pathophysiology of the disease, different medical complications might arise, ranging from infections to psychiatric effects such as mania, which can pose harm to the patient or undermine their autonomy. Explantation, then, may be the favored route to alleviate these complications. However, some authors have highlighted the fact that explantation for medical indications may also involve an element of coercion. This so-called “forced explantation” can override the patient’s autonomy and violate their “bodily integrity,” raising serious ethical concerns. A critical note is that this perspective, as proposed by Gilbert (2013), seems to disregard the premise that physicians act according to the principle of “do no unnecessary harm”—or *non-maleficence*. Additionally, our study found issues regarding patients’ rights to their implants, underscoring the tension between medical necessity and respect for personal agency. Overall, these considerations reveal a critical dimension often overlooked in harm-benefit analyses: the need to safeguard a patient’s autonomy, even when explantation is medically justified.

Moreover, these findings call into question the comprehensiveness of the procedure of informed consent in trials involving neural implants. While informed consent is intended to protect patients’ autonomy, the complexity of these interventions—coupled with the potential for unforeseen medical complications or coercion surrounding explantation—suggests that patients may not fully comprehend the long-term implications of their participation. For example, the possibility of “unwanted” explantation might not be adequately conveyed during the consent procedure, leaving patients unaware of the potential loss of control over their bodies and DBS and RNS devices. Sankary et al. (2023) interviewed study participants who were indeed blinded by *therapeutic misconception*: they reported to hope for symptom relief and “[…] did not really focus on the end.” From a legal perspective, neurorights were suggested to provide legal boundaries in neurodevice research (Gilbert et al., 2023). These rights view implanted persons as a new legal personhood and enforce researchers to adapt their informed consent procedures as a continuous process, rather than conflating consent at the pre-implantation phase with consent to explantation when implanted. Bublitz and Gilbert (2023) further drew on neurorights and the ethical dilemmas in neural implant research participants that come to face explantation. They presented additional questions that may arise from explantation of a life-altering neural implant: (1) whether participants can refuse the explantation and keep devices; (2) whether they are entitled to continuous support by manufacturers; (3) whether they may invoke human rights in support of their cause as suggested in the literature. The example of neurorights and additional legal analyses serve as a stepping stone in development of a legal framework regarding neurodevice treatment, including decision-making on explantation and regard to users’ autonomy and rights.

### 4.2 Strengths and limitations

Finally, to our knowledge, this systematic review is the first to cover multiple domains in explantation of neurodevices. The strength lies in its broad scope and inclusion of a hundred studies with various insights on the subject. To widen the scope, the search was carried out in various databases not limited to medical databases, such as PsychINFO and Philosopher’s Index. For a systematic thematic analysis, we chose the novel IEEE Neuroethics framework (IEEE brain, n.d.) to delineate the different considerations in explantation. One of our reasons to use this framework is because it is a “live” document that is created and curated by an international interdisciplinary group of experts, including physicians, engineers, and ethicists (IEEE brain, n.d.). These different perspectives are crucial to understand the various considerations in explantation of neurodevices. As multiple stakeholders are involved, such a multidisciplinary approach and wide scope is needed to shine light on the complex issues in neural implant explantation. Additionally, the categories—ethical, legal, and sociocultural—are easy to use and accessible to a non-ethics audience which is necessary for its further cultivation on the issue of explantation. A limitation of this study is a consequence of the inclusion of qualitative and ethical studies, for which a standardized bias assessment tool does not exist. To minimize bias, we have used the data extraction method inspired by Strech and Sofaer (2012) which couples reason types (considerations) with exact passages in the articles. Subsequent categorization according to the IEEE Neuroethics framework (IEEE brain, n.d.) was done by two independent authors to further decrease the risk of bias. Another limitation was the overall heterogeneity in the publications, such as type of technology used and study design. Data was also skewed towards DBS—perhaps due to the simple fact that DBS has been studied for longer and more thoroughly, especially in its application in Parkinson’s disease (Cavallieri et al., 2024). Overall, this type of data extraction and categorization yielded a comprehensive overview of the literature on neural device explantation.

## 5 Conclusion

Neural implants have a broad range of applications ranging from Parkinson’s disease to locked-in syndrome. Their increasing application raises questions on decision-making in explantation in medical and research settings. Our article provides a systematic review of the considerations in explantation of neurodevices categorized using the IEEE BRAIN Neuroethics framework (IEEE brain, n.d.). We have identified several considerations including medical complications, changes in cognition and behavior, emotional well-being, a lack of benefit, financial issues, post-trial considerations, neurorights, cosmetic-related, and identity. While the literature currently focusses on medical complications, these findings highlight the multifaceted nature of neural implant explantation and the need for interdisciplinary approaches to address the associated challenges. A major research opportunity lies in creating a standardized definition of explantation to compare outcomes worldwide. As this research demonstrates, there are anecdotal incidents where neural implants seem to have a relationship with life-threatening symptoms such as depersonalization and suicidality, but the current lack of standardization makes it difficult to draw comparisons between such cases. International consensus on definitions of explantation allow for comparability in medical studies and can aid decision-making in difficult ethical scenarios. In a similar fashion, studies are encouraged to include pre- and post-implantation psychological assessments when researching neural implants. Multiple points of assessment and a substantial follow-up period can give insights on the impact of device implantation and explantation, especially in patient groups with psychiatric comorbidities (such as DBS for patients with depression). Following the IEEE BRAIN Neuroethics framework (IEEE brain, n.d.), our review also demonstrates a lack of studies on surveillance and privacy considerations in neural device explantation. Future endeavors can focus on these themes and shine light on questions around data storage, collection, usage, and analysis. Overall, the IEEE BRAIN Neuroethics framework (IEEE brain, n.d.) has shown to provide a comprehensive overview and researchers are encouraged to use this multidisciplinary approach on ethical issues in neural implants research. This study serves as a steppingstone in standardizing neural implant research by collecting ethical, sociocultural, and legal considerations in neural device explantation as well the current issues in the field.

## Data Availability

The original contributions presented in the study are included in the article/Supplementary material, further inquiries can be directed to the corresponding author.

## Supplementary material

Supplementary Data 1 Search strategy.

Supplementary Data Sheet 1 General information.

Supplementary Data sheet 2 Overview of ethical, legal, and sociocultural considerations in explantation.
