# Supplementary material for: Ethical, legal, and sociocultural considerations in neural device explantation: a systematic review
Source: Front Neurosci. 2025 Nov 3;19:1568800. doi: 10.3389/fnins.2025.1568800 (PMC12621010; doi:10.3389/fnins.2025.1568800)
Supplement: Supplementary Data sheet 2 — Overview of ethical, legal, and sociocultural considerations in explantation. [file Supplementary_file_1.docx]

Supplementary Material

# Supplementary Data

*Search Strategy*

(("Brain-Computer Interfaces"[Mesh] OR "Auditory Brain Stem Implants"[Mesh] OR "Cochlear Implants"[Mesh] OR "Deep Brain Stimulation"[Mesh] OR "Implantable Neurostimulators"[Mesh] OR "Neural Prostheses"[Mesh] OR "brain computer interface"[tw] OR "brain computer interfaces"[tw] OR "brain electrode"[tw] OR "brain electrodes"[tw] OR "brain implant"[tw] OR "brain implants"[tw] OR "brain modulation"[tw] OR "brain modulator"[tw] OR "brain modulators"[tw] OR "brain stimulation"[tw] OR "brain stimulator"[tw] OR "brain stimulators"[tw] OR "brain-computer interface"[tw] OR "brain-computer interfaces"[tw] OR "central nervous system device"[tw] OR "central nervous system devices"[tw] OR "central nervous system electrode"[tw] OR "central nervous system electrodes"[tw] OR "central nervous system implant"[tw] OR "central nervous system implants"[tw] OR "central nervous system modulation"[tw] OR "central nervous system modulator"[tw] OR "central nervous system modulators"[tw] OR "central nervous system stimulator"[tw] OR "central nervous system stimulators"[tw] OR "cerebral device"[tw] OR "cerebral devices"[tw] OR "cerebral electrode"[tw] OR "cerebral electrodes"[tw] OR "cerebral implant"[tw] OR "cerebral implants"[tw] OR "CNS implant"[tw] OR "CNS implants"[tw] OR "CNS stimulator"[tw] OR "CNS stimulators "[tw] OR "cochlear device"[tw] OR "cochlear devices"[tw] OR "cochlear implant"[tw] OR "cochlear implants"[tw] OR "computer central nervous system"[tw] OR "deep brain stimulation"[tw] OR "epilepsy device"[tw] OR "epilepsy devices"[tw] OR "in-ear device"[tw] OR "in-ear devices"[tw] OR "in-ear implant"[tw] OR "in-ear implants"[tw] OR "intracerebral device"[tw] OR "intracerebral devices"[tw] OR "intracerebral electrode"[tw] OR "intracerebral electrodes"[tw] OR "intracerebral implant"[tw] OR "intracerebral implants"[tw] OR "nerve electrode"[tw] OR "nerve electrodes"[tw] OR "nerve stimulator"[tw] OR "nerve stimulators"[tw] OR "neural device"[tw] OR "neural devices"[tw] OR "neural stimulator"[tw] OR "neural stimulators"[tw] OR "neurodevice"[tw] OR "neurodevices"[tw] OR "neurologic implant"[tw] OR "neurologic implants"[tw] OR "neurological implant"[tw] OR "neurological implants"[tw] OR "neuron stimulator"[tw] OR "neuron stimulators"[tw] OR "neuronal device"[tw] OR "neuronal devices"[tw] OR "neuronal electrode"[tw] OR "neuronal electrodes"[tw] OR "neuronal implant"[tw] OR "neuronal implants"[tw] OR "neuronal stimulator"[tw] OR "neuronal stimulators"[tw] OR "neuroprosthesis"[tw] OR "neuroprostheses"[tw] OR "neuroprosthetic"[tw] OR "neuroprosthetics"[tw] OR "neuro-prosthetic"[tw] OR "neuro-prosthetics"[tw] OR "neuro-prosthesis"[tw] OR "neuro-prostheses"[tw] OR "neuroprothesis"[tw] OR "neurostimulator"[tw] OR "neurostimulators"[tw] OR "neurosurgical implant"[tw] OR "neurosurgical implants"[tw] OR "retinal device"[tw] OR "retinal devices"[tw] OR "retinal implant"[tw] OR "retinal implants"[tw] OR "spinal device"[tw] OR "spinal devices"[tw] OR "spinal electrode"[tw] OR "spinal electrodes"[tw] OR "spinal implant"[tw] OR "spinal implants"[tw] OR "spine computer interface"[tw] OR "spine computer interfaces"[tw] OR "spine electrode"[tw] OR "spine electrodes"[tw] OR "spine modulation"[tw] OR "spine modulators"[tw] OR "spine modulator"[tw] OR "spine stimulator"[tw] OR "spine stimulators"[tw] OR "vagal nerve stimulator"[tw] OR "vagal nerve stimulators"[tw] OR "vagus nerve stimulator"[tw] OR "vagus nerve stimulators"[tw] OR "Auditory Prostheses"[tw] OR "Auditory Prosthesis"[tw] OR "Brain Machine Interface"[tw] OR "Brain Machine Interfaces"[tw] OR "Brain Stem Implant"[tw] OR "Brain Stem Implants"[tw] OR "Cochlear Prostheses"[tw] OR "Cochlear Prosthesis"[tw] OR "Nerve Stimulation Electrode"[tw] OR "Nerve Stimulation Electrodes"[tw] OR "functional neurosurgery"[tw] OR "brain computer interface"[title/abstract:~3] OR "brain computer interfaces"[title/abstract:~3] OR "brain electrode"[title/abstract:~3] OR "brain electrodes"[title/abstract:~3] OR "brain implant"[title/abstract:~3] OR "brain implants"[title/abstract:~3] OR "brain modulation"[title/abstract:~3] OR "brain modulator"[title/abstract:~3] OR "brain modulators"[title/abstract:~3] OR "brain stimulation"[title/abstract:~3] OR "brain stimulator"[title/abstract:~3] OR "brain stimulators"[title/abstract:~3] OR "brain computer interface"[title/abstract:~3] OR "brain computer interfaces"[title/abstract:~3] OR "central nervous system device"[title/abstract:~3] OR "central nervous system devices"[title/abstract:~3] OR "central nervous system electrode"[title/abstract:~3] OR "central nervous system electrodes"[title/abstract:~3] OR "central nervous system implant"[title/abstract:~3] OR "central nervous system implants"[title/abstract:~3] OR "central nervous system modulation"[title/abstract:~3] OR "central nervous system modulator"[title/abstract:~3] OR "central nervous system modulators"[title/abstract:~3] OR "central nervous system stimulator"[title/abstract:~3] OR "central nervous system stimulators"[title/abstract:~3] OR "cerebral device"[title/abstract:~3] OR "cerebral devices"[title/abstract:~3] OR "cerebral electrode"[title/abstract:~3] OR "cerebral electrodes"[title/abstract:~3] OR "cerebral implant"[title/abstract:~3] OR "cerebral implants"[title/abstract:~3] OR "CNS implant"[title/abstract:~3] OR "CNS implants"[title/abstract:~3] OR "CNS stimulator"[title/abstract:~3] OR "CNS stimulators "[title/abstract:~3] OR "cochlear device"[title/abstract:~3] OR "cochlear devices"[title/abstract:~3] OR "cochlear implant"[title/abstract:~3] OR "cochlear implants"[title/abstract:~3] OR "computer central nervous system"[title/abstract:~3] OR "deep brain stimulation"[title/abstract:~3] OR "epilepsy device"[title/abstract:~3] OR "epilepsy devices"[title/abstract:~3] OR "in ear device"[title/abstract:~3] OR "in ear devices"[title/abstract:~3] OR "in ear implant"[title/abstract:~3] OR "in ear implants"[title/abstract:~3] OR "intracerebral device"[title/abstract:~3] OR "intracerebral devices"[title/abstract:~3] OR "intracerebral electrode"[title/abstract:~3] OR "intracerebral electrodes"[title/abstract:~3] OR "intracerebral implant"[title/abstract:~3] OR "intracerebral implants"[title/abstract:~3] OR "nerve electrode"[title/abstract:~3] OR "nerve electrodes"[title/abstract:~3] OR "nerve stimulator"[title/abstract:~3] OR "nerve stimulators"[title/abstract:~3] OR "neural device"[title/abstract:~3] OR "neural devices"[title/abstract:~3] OR "neural stimulator"[title/abstract:~3] OR "neural stimulators"[title/abstract:~3] OR "neurologic implant"[title/abstract:~3] OR "neurologic implants"[title/abstract:~3] OR "neurological implant"[title/abstract:~3] OR "neurological implants"[title/abstract:~3] OR "neuron stimulator"[title/abstract:~3] OR "neuron stimulators"[title/abstract:~3] OR "neuronal device"[title/abstract:~3] OR "neuronal devices"[title/abstract:~3] OR "neuronal electrode"[title/abstract:~3] OR "neuronal electrodes"[title/abstract:~3] OR "neuronal implant"[title/abstract:~3] OR "neuronal implants"[title/abstract:~3] OR "neuronal stimulator"[title/abstract:~3] OR "neuronal stimulators"[title/abstract:~3] OR "neuro prosthetic"[title/abstract:~3] OR "neuro prosthetics"[title/abstract:~3] OR "neuro prosthesis"[title/abstract:~3] OR "neuro prostheses"[title/abstract:~3] OR "neurosurgical implant"[title/abstract:~3] OR "neurosurgical implants"[title/abstract:~3] OR "retinal device"[title/abstract:~3] OR "retinal devices"[title/abstract:~3] OR "retinal implant"[title/abstract:~3] OR "retinal implants"[title/abstract:~3] OR "spinal device"[title/abstract:~3] OR "spinal devices"[title/abstract:~3] OR "spinal electrode"[title/abstract:~3] OR "spinal electrodes"[title/abstract:~3] OR "spinal implant"[title/abstract:~3] OR "spinal implants"[title/abstract:~3] OR "spine computer interface"[title/abstract:~3] OR "spine computer interfaces"[title/abstract:~3] OR "spine electrode"[title/abstract:~3] OR "spine electrodes"[title/abstract:~3] OR "spine modulation"[title/abstract:~3] OR "spine modulators"[title/abstract:~3] OR "spine modulator"[title/abstract:~3] OR "spine stimulator"[title/abstract:~3] OR "spine stimulators"[title/abstract:~3] OR "vagal nerve stimulator"[title/abstract:~3] OR "vagal nerve stimulators"[title/abstract:~3] OR "vagus nerve stimulator"[title/abstract:~3] OR "vagus nerve stimulators"[title/abstract:~3] OR "Auditory Prostheses"[title/abstract:~3] OR "Auditory Prosthesis"[title/abstract:~3] OR "Brain Machine Interface"[title/abstract:~3] OR "Brain Machine Interfaces"[title/abstract:~3] OR "Brain Stem Implant"[title/abstract:~3] OR "Brain Stem Implants"[title/abstract:~3] OR "Cochlear Prostheses"[title/abstract:~3] OR "Cochlear Prosthesis"[title/abstract:~3] OR "Nerve Stimulation Electrode"[title/abstract:~3] OR "Nerve Stimulation Electrodes"[title/abstract:~3] OR "functional neurosurgery"[title/abstract:~3]) AND ("Device Removal"[Mesh] OR "deimplant"[tw] OR "deimplant*"[tw] OR "deimplantation"[tw] OR "deimplants"[tw] OR "device removal"[tw] OR "exit strategiees"[tw] OR "exit strategy"[tw] OR "explant"[tw] OR "explantat*"[tw] OR "explantation"[tw] OR "explants"[tw] OR "neurosurgical removal"[tw] OR "prosthetic removal"[tw] OR "prosthetics removal"[tw] OR "prothesis removal"[tw] OR "protheses removal"[tw] OR "implants removal"[tw] OR "implant removal"[tw] OR "removal"[tw] OR "remove"[tw] OR "remov*"[tw] OR "surgical removal"[tw] OR "device removal"[title/abstract:~3] OR "exit strategiees"[title/abstract:~3] OR "exit strategy"[title/abstract:~3] OR "neurosurgical removal"[title/abstract:~3] OR "prosthetic removal"[title/abstract:~3] OR "prosthetics removal"[title/abstract:~3] OR "protheses removal"[title/abstract:~3] OR "implants removal"[title/abstract:~3] OR "implant removal"[title/abstract:~3] OR "surgical removal"[title/abstract:~3]) AND ("Ethics"[Mesh] OR "ethics"[Subheading] OR "Decision Theory"[Mesh] OR "Decision Support Techniques"[Mesh] OR "Clinical Decision-Making"[Mesh] OR "Decision Making"[Mesh] OR "Duty to Recontact"[Mesh] OR "Duty to Warn"[Mesh] OR "Moral Obligations"[Mesh] OR "Morals"[Mesh] OR "decision"[tw] OR "decisions"[tw] OR "decision*"[tw] OR "decisionmaking"[tw] OR "decision making"[tw] OR "duties"[tw] OR "duty"[tw] OR "ethic"[tw] OR "ethical"[tw] OR "ethical research"[tw] OR "ethics"[tw] OR "ethic*"[tw] OR "human research ethics"[tw] OR "moral obligation"[tw] OR "moral obligations"[tw] OR "morals"[tw] OR "morality"[tw] OR "morals"[tw] OR "obligation"[tw] OR "obligations"[tw] OR "research ethics"[tw] OR "decision making"[title/abstract:~3] OR "ethical research"[title/abstract:~3] OR "human research ethics"[title/abstract:~3] OR "moral obligation"[title/abstract:~3] OR "moral obligations"[title/abstract:~3] OR "research ethics"[title/abstract:~3] OR "bioethical research"[title/abstract:~3] OR "moral policy"[title/abstract:~3] OR "moral policies"[title/abstract:~3] OR "bioethic"[tw] OR "bioethical"[tw] OR "bioethical research"[tw] OR "bioethics"[tw] OR "bioethic*"[tw] OR "moral policy"[tw] OR "moral policies"[tw] OR "metaethics"[tw] OR "metaethic"[tw] **OR "human rights"[tw]** **OR "Human Rights"[Mesh] OR "Human Rights Abuses"[Mesh] OR "Social Behavior"[Mesh] OR (("role"[tw] OR "roles"[tw]) AND ("Surgeons"[mesh] OR "Neurosurgeons"[mesh] OR "Surgeon"[tw] OR "Surgeons"[tw] OR "Neurosurgeon"[tw] OR "Neurosurgeons"[tw]))**))
